# Evaluating the Safety of Simultaneous Intracranial Electroencephalography and Functional Magnetic Resonance Imaging Acquisition Using a 3 Tesla Magnetic Resonance Imaging Scanner

**DOI:** 10.3389/fnins.2022.921922

**Published:** 2022-06-23

**Authors:** Yuya Fujita, Hui Ming Khoo, Miki Hirayama, Masaaki Kawahara, Yoshihiro Koyama, Hiroyuki Tarewaki, Atsuko Arisawa, Takufumi Yanagisawa, Naoki Tani, Satoru Oshino, Louis Lemieux, Haruhiko Kishima

**Affiliations:** ^1^Department of Neurosurgery, Osaka University Graduate School of Medicine, Suita, Japan; ^2^Department of Radiology, Osaka University Hospital, Suita, Japan; ^3^Department of Radiology, Osaka University Graduate School of Medicine, Suita, Japan; ^4^Department of Clinical and Experimental Epilepsy, UCL Queen Square Institute of Neurology, London, United Kingdom

**Keywords:** MRI, simultaneous intracranial EEG-fMRI, subdural electrode, depth electrode, 3 tesla, fMRI, epilepsy, safety

## Abstract

**Background:**

The unsurpassed sensitivity of intracranial electroencephalography (icEEG) and the growing interest in understanding human brain networks and ongoing activities in health and disease have make the simultaneous icEEG and functional magnetic resonance imaging acquisition (icEEG-fMRI) an attractive investigation tool. However, safety remains a crucial consideration, particularly due to the impact of the specific characteristics of icEEG and MRI technologies that were safe when used separately but may risk health when combined. Using a clinical 3-T scanner with body transmit and head-receive coils, we assessed the safety and feasibility of our icEEG-fMRI protocol.

**Methods:**

Using platinum and platinum-iridium grid and depth electrodes implanted in a custom-made acrylic-gel phantom, we assessed safety by focusing on three factors. First, we measured radio frequency (RF)-induced heating of the electrodes during fast spin echo (FSE, as a control) and the three sequences in our icEEG-fMRI protocol. Heating was evaluated with electrodes placed orthogonal or parallel to the static magnetic field. Using the configuration with the greatest heating observed, we then measured the total heating induced in our protocol, which is a continuous 70-min icEEG-fMRI session comprising localizer, echo-planar imaging (EPI), and magnetization-prepared rapid gradient-echo sequences. Second, we measured the gradient switching-induced voltage using configurations mimicking electrode implantation in the frontal and temporal lobes. Third, we assessed the gradient switching-induced electrode movement by direct visual detection and image analyses.

**Results:**

On average, RF-induced local heating on the icEEG electrode contacts tested were greater in the orthogonal than parallel configuration, with a maximum increase of 0.2°C during EPI and 1.9°C during FSE. The total local heating was below the 1°C safety limit across all contacts tested during the 70-min icEEG-fMRI session. The induced voltage was within the 100-mV safety limit regardless of the configuration. No gradient switching-induced electrode displacement was observed.

**Conclusion:**

We provide evidence that the additional health risks associated with heating, neuronal stimulation, or device movement are low when acquiring fMRI at 3 T in the presence of clinical icEEG electrodes under the conditions reported in this study. High specific absorption ratio sequences such as FSE should be avoided to prevent potential inadvertent tissue heating.

## Introduction

There are several modalities used to analyze brain activity. Scalp electroencephalography (EEG) is a simple method for measuring brain signals with high temporal resolution. However, EEG source localization is generally difficult for the superficial activity that EEG can actually detect, in particular if the generator is widespread and complex. Functional magnetic resonance imaging (fMRI) focuses on the changes in cerebral blood flow, using blood oxygen level-dependent contrast for probing brain activity, regardless of the coverage and indifference to the extent and complexity of the source. By effectively combining their advantages, simultaneous EEG and fMRI measurement (EEG-fMRI) is an attractive investigation tool for those who are interested in understanding the relationship between the two modalities. For example, EEG-fMRI is used in epilepsy to understand the mechanisms underlying the generation of epileptic activities, spontaneous brain activities that are unpredictable (Gotman et al., 2006; Khoo et al., 2017). It is also used in the field of neuroscience to study the hemodynamic correlates of event-related potentials and to study neurofeedback (Mele et al., 2019). However, activities in the high-frequency band, markers of most cognitive neuronal activities, are difficult to record on scalp EEG, and the information gained from these activities are also limited for the following reasons. (1) Spectral power follows a 1/f distribution across frequencies and thus high gamma activities are generally lower in amplitude than that of low frequency activities (Baranauskas et al., 2012; Jaspers-Fayer et al., 2012; Li et al., 2019). (2) The skull further attenuates the amplitude of EEG, generally resulting in a low signal-to-noise ratio especially in the high-frequency band. (3) High-frequency activities overlap the spectral bandwidth of muscle activities. (4) Scalp EEG has low sensitivity to activity generated deep in the brain as it detects mostly neocortical activity (Wennberg et al., 2011). Intracranial EEG (icEEG) electrodes, implanted to delineate the epileptogenic zone of patients with drug-resistant epilepsy prior to resection, provide increased sensitivity to activities in the high-frequency range, while allowing detection of low amplitude activities in a lower frequency range (i.e., epileptiform activities). Hence, the sensitivity of icEEG acquired simultaneously with fMRI provides a good opportunity to study neuronal activities more substantively (Vulliemoz et al., 2011; Cunningham et al., 2012; Tehrani et al., 2021).

There are three potential hazards associated with the introduction of icEEG electrodes to the magnetic resonance imaging (MRI) environment (Carmichael et al., 2010; Boucousis et al., 2012): radiofrequency (RF)-induced heating of brain tissue surrounding the electrodes, in which the temperature increase of a device is conservatively limited to within 1°C of the surrounding tissue according to current safety standards (IEC, 2015); neural stimulation or tissue damage caused by induced currents in low-resistance circuits generated by magnetic field fluctuations such as gradient switching (Georgi et al., 2004; Wang et al., 2018), in which a voltage exceeding 100 mV at a frequency less than 10 kHz can cause neural stimulation (Georgi et al., 2004); and tissue damage due to uncontrolled electrode movement caused by forces or torques induced by the static or dynamic magnetic field on the electrode.

Although deep brain stimulation (DBS) electrode (MRI-conditional for 3 T) and icEEG electrodes from a specific manufacturer (DIXI medical, MRI-conditional for 1.5–3 T) are allowed for MRI under the restrictive guidelines of manufacturers, most commercial icEEG electrodes have yet to be formally approved for MRI (Ciumas et al., 2014; Hawsawi et al., 2017). Nevertheless, structural imaging of icEEG electrodes has been well documented in both clinical and research settings without adverse events at 1.5 T (Davis et al., 1999; Carmichael et al., 2007, 2008; Larson et al., 2008; Nazzaro et al., 2010; Weise et al., 2010; Vulliemoz et al., 2011; Zrinzo et al., 2011; Hawsawi et al., 2017, 2020; Erhardt et al., 2018; Hall and Khoo, 2018; Yazdani et al., 2021). Imaging of DBS electrodes at 3 T has also been documented in 10 patients with a mild temperature increase and concluded to be potentially safe (Sammartino et al., 2017). Due to the potential of increased risk, clinical imaging of icEEG electrodes at 3 T has never been documented (Hawsawi et al., 2017). Nonetheless, simultaneous acquisition of icEEG and fMRI (icEEG-fMRI) has been documented in a few human studies at 1.5 T performed in two institutions (Vulliemoz et al., 2011; Carmichael et al., 2012; Chaudhary et al., 2016; Ridley et al., 2017; Saignavongs et al., 2017; Liu et al., 2022) and at 3 T in another (Aghakhani et al., 2015; Tehrani et al., 2021), using a local imaging protocol developed in each institution for research, without reports of significant adverse events to date. Hence, icEEG-fMRI appears to pose a low health risk to patients provided that site-specific precautions are taken.

However, findings of the icEEG-fMRI safety studies that justified the choice of MRI field strength by two of the aforementioned institutions, appeared to be conflicting: significant RF-induced heating at 3 T was documented in one (Carmichael et al., 2008) but not in the other (Boucousis et al., 2012) even though the same type of electrodes (platinum-iridium electrodes from Ad-Tech Medical, Racine, WI, United States) has been used. This difference can be attributed to various factors given the difference in the equipment and conditions used in each institution and thus imaging of icEEG electrodes at 3 T remains controversial. The logical conclusion on the safety of any icEEG-fMRI acquisition is that it is dependent on specific conditions and a careful assessment must be made for any significant deviation from the tested conditions, including electrode implantation and wiring configurations, scanner type and field strength, type of RF coil (body or head transmit), MRI scanning protocol and sequences (Carmichael et al., 2008, 2010).

In this study, we assessed the feasibility of performing icEEG-fMRI over the entire course of a typical prolonged acquisition that lasted for approximately 70 min in a GE Signa Architect UPG 3 T MRI scanner with a body transmit head-receive coil, using grid and depth electrodes commercially available in Japan. To this effect, following previous work on the safety of icEEG-fMRI (Carmichael et al., 2008, 2010; Boucousis et al., 2012), we performed temperature, voltage, and electrode movement measurements on a standard gel and acrylic phantom. If proven feasible, the findings may serve as a guidance for developing clinical combined icEEG-fMRI protocols at 3 T and provide another piece of evidence regarding the feasibility of icEEG-fMRI at 3 T, an MRI field strength that is becoming the standard in clinical and research settings due to the improved signal-to-noise ratio that it offers.

## Materials and Methods

### Phantom Preparation

**FIGURE 1:**
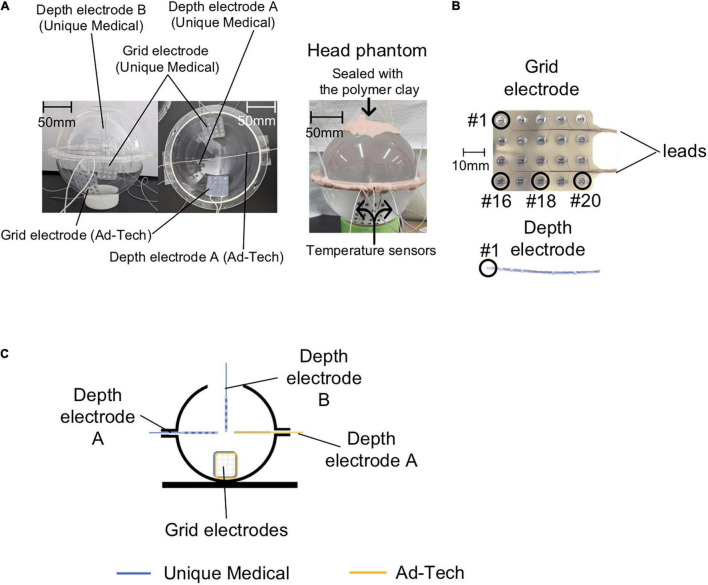
Phantom preparation. **(A)** Left panel: Photos of the empty acrylic shells are shown here to clarify the position of each electrode in the head phantom. Two Unique Medical depth electrodes were placed in the acrylic shells: one was quasi-perpendicular (depth electrode A) and the other was quasi-parallel (depth electrode B) to the grid electrode. Only one Ad-Tech depth electrode was implanted, which was quasi-perpendicular to the grid electrode (depth electrode A). Right panel: photo of the head phantom that was ready for use in experiments of both the temperature and voltage measurements. **(B)** Electrode contact numbering. In the case of grid electrodes, #1 represents the contact at the right corner and the most distal from the leads, and #20 represents the contact at the left corner and the most proximal to the leads. In the case of depth electrodes, #1 represents the contact at the tip. **(C)** The head phantom as represented in a two-dimensional line drawing.

To better reflect actual icEEG-fMRI acquisitions, we used a phantom made of two elements: a spherical head and a rectangular torso with electrical conductivity and thermal characteristics similar to those of human tissue. The head part of the phantom was made by combining two custom-made hollow hemispherical acrylic shells (diameter = 150 mm), in which one of them had an opening (diameter = 50 mm) on the top. We used a commercially available polypropylene box (42 L: width 362 mm, length 617 mm, height 185 mm) as the container for the body part of the phantom. The containers were then filled up with a semi-liquid gel comprising distilled water, poly-acrylic acid partial sodium salt (A9799; Sigma-Aldrich, St. Louis, MO, United States) and sodium chloride (008-71265; Kishida Chemical Co., Ltd., Osaka, Japan). The gel had an electrical conductivity of 0.26 S m^–1^ and limited thermal convection to mimic those of human tissue (Carmichael et al., 2010). The gels for the head and torso were made by adding sodium chloride (1.4 g for the head, 28 g for the torso) to distilled water (2 L for the head, 40 L for the torso) heated to 40°C to prevent air bubbles forming in the gel. Then we gradually added poly-acrylic acid partial sodium salt (16 g for the head, 320 g for the torso) while slowly stirring the solutions. We pre-implanted the electrodes in the head phantom from the top opening and from the gap between the two hemispheres. To mimic how the electrodes are placed in real human subjects, we fixed the grid electrodes tangential to the inner surface of the acrylic container with the electrode contact exposure facing the center, and placed the depth electrodes perpendicular to the surface of the acrylic container with all of the contacts embedded within the gel (Figure 1A). Then the gap was sealed with a polymer clay made of polyvinyl (Super Sculpey^®^ Beige, Polyform Products Company, Elk Grove Village, IL, United States).

We tested two types of commercially available intracranial grid and depth electrodes: the first type was from Unique Medical (Tokyo, Japan), which we are currently using at our center; the second type was from Ad-Tech Medical (Racine, WI, United States), which has been used in previous safety studies (Carmichael et al., 2010; Boucousis et al., 2012). The grid electrodes have 20 disk-shaped contacts, and the depth electrodes have six contacts (Figure 1B) connected via lead wires to connector terminals. Table 1 summarizes the composition and dimension of each electrode type.

**TABLE 1:** Materials and dimension of the electrodes used.

	Unique Medical Co. Ltd.	Ad-Tech Medical Instrument Co.
		
Grid electrode	UZN C1-20-05-10-2-A	FG20C-SP10X-000
***Dimension***		
Number of contacts	20 (4 × 5)	20 (4 × 5)
Center-to-center contact spacing (mm)	10	10
Contact diameter/exposure (mm)	5/3	4/2.3
Lead wire diameter (mm)	0.08	0.0635
Total length (mm)	465	430
***Materials***		
Contacts	Platinum	Platinum–iridium
Imbedding sheet	Silicon	Silicon
Lead wire	Stainless steel	Stainless steel
Lead tubing	Silicon	Silicon
Connector terminal	Stainless steel	Nickel–chromium

**Depth Electrode**	**UZN D4-06-054-151-101-A**	**SD06R-SP10X-000**

***Dimension***		
Number of contacts	6	6
Center-to-center contact spacing (mm)	5 between the first four contacts from the tip 15 between contact #4 and #5 10 between contact #5 and #6	10
Contact length (mm)	1.0	1.32
Electrode diameter (mm)	1.5	1.1
Lead wire diameter (mm)	0.08	0.0635
Total length (mm)	450	380
***Materials***		
Contacts	Platinum	Platinum
Lead wire	Platinum	Nickel–chromium
Lead tubing	Silicon	Polyurethane
Connector terminal	Stainless steel	Nickel–chromium

The Unique Medical electrodes were implanted on one side and the Ad-Tech electrodes on the contralateral in the same head phantom to allow fair comparisons between the two types of electrodes. The two grid electrodes were placed at the bottom side of the acrylic container. For Unique Medical, two depth electrodes were implanted: one was placed quasi-perpendicular (depth electrode A) and one quasi-parallel (depth electrode B) to the grid electrode (Figures 1A,C). For Ad-Tech, only one depth electrode was implanted (depth electrode A) quasi-perpendicular to the grid electrode (Figures 1A,C). In total, two grid electrodes and three depth electrodes were placed in the head phantom. Since the head phantom was not attached to the body phantom, we rotated the head phantom accordingly to achieve the configurations needed in each experiment during either the temperature or voltage measurement (see “*The Experiments: Electrode and phantom configurations”* section below for details).

### Temperature Measurement Methodology

**FIGURE 2:**
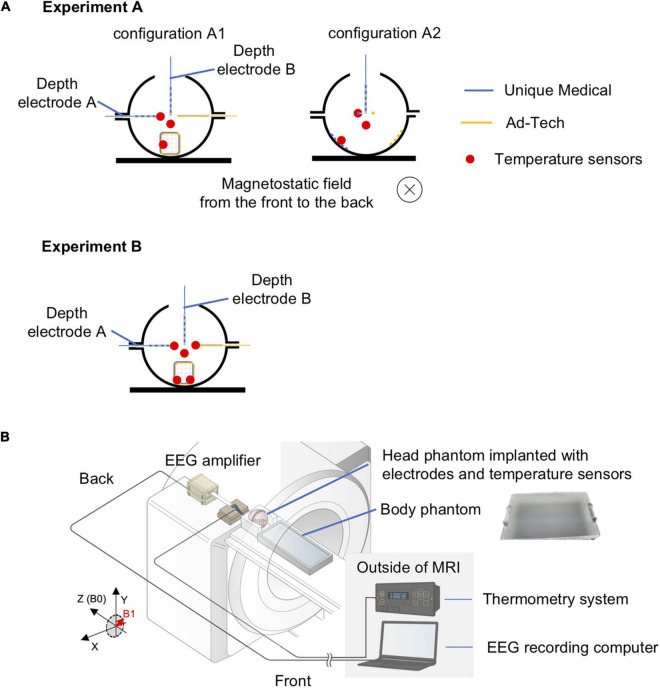
Experimental setup for temperature measurement of radiofrequency-induced heating. **(A)** Orientation of the head phantom and the location of temperature sensors during experiment A (upper panel) and experiment B (lower panel). In experiment A, two conditions were tested: configuration A1 – electrodes placed orthogonal to the direction of the static magnetic field; configuration A2 – electrodes placed parallel to the direction of the static magnetic field. The blue electrodes were Unique Medical electrodes, and the yellow electrodes were Ad-Tech. The location of a temperature sensor was indicated as red dot. The red dot distanced from all electrodes represents the sensor placed within the gel to serve as control. Experiment B was performed twice: two sensors were placed on Unique Medical electrodes during the first and on Ad-Tech electrodes during the second time. **(B)** Layout of the phantoms and all of the equipment during experiments for temperature measurement. The head and body phantoms were placed in the magnetic resonance imaging (MRI) scanner with the head at the isocenter to emulate an actual simultaneous acquisition of icEEG and fMRI (icEEG-fMRI). The electrodes implanted in the head phantom were connected to the EEG amplifier placed at the “back” of the MRI bore, with exactly the same configuration as an actual icEEG-fMRI acquisition in human subject. The electrodes were secured using memory foam cushions in the head coil and cables were secured using sandbags to prevent movements resulted from mechanical vibration of the MRI scanners during the experiment. The fiber optic MRI-compatible temperature sensors, fixed to the intracranial electrodes, were connected via optical fibers to the thermometry system placed outside the MRI in the control room.

The temperature was measured continuously and simultaneously from four locations using a four-channel, fiber-optic thermometry system (MultiSens; Opsens Solutions, Quebec, QC, Canada) connected to four fiber optic MRI-compatible temperature sensors (OTP-M; Opsens Solutions, Quebec, QC, Canada, accuracy ± 0.30°C) with a sampling rate of 1/1.4 s. We placed the temperature sensors at the electrode tip, the location most likely to demonstrate the largest temperature change as shown in previous studies (Boucousis et al., 2012; Carmichael et al., 2012), as follows: the most distal contact (#1) of the 6-contact depth electrode A, the corner or edge of the grid (#16 or #18) (Figure 1B). A temperature sensor was placed at the middle of the head phantom, distanced from all electrodes to serve as a control (Figure 2A). In the case of grid electrodes, the temperature sensors were laid in a transverse position on the surface of the disk contact and held in place using a silk suture through the silicon. In the case of depth electrodes, the sensor was laid parallel, and tied to the electrode contact using a silk thread. Then the sensors were connected via optical fibers to the thermometry system placed outside the MRI room (Figure 2B). To mimic concurrent icEEG-fMRI acquisitions, the electrodes were connected to the EEG amplifiers (Brain Products, Gilching, Germany) via connector cables (180 cm, Tech-Attach Connection System; Ad-Tech Medical) attached to a 64-channel touch-proof electrode input box (EIB64; Brain Products). The connector cables were folded (with 10 cm folds, as previously reported; Carmichael et al., 2012) and placed straight at the center of the MRI bore to minimize the MRI-induced current. To avoid movement due to machinery-induced vibration, the external portion of the leads of the electrodes (between the phantom head and the connector cables) were sandwiched between memory foam cushions that were placed around the phantom head inside the head coil. MRI-compatible sandbags were used to immobilize the cables between the head coil and the EEG amplifiers, which were connected via optic fibers to the recording computer placed outside the MRI room. The signals from the implanted electrodes were recorded during temperature measurements to mimic an actual icEEG-fMRI acquisition on human subjects.

### Voltage Measurement Methodology

**FIGURE 3:**
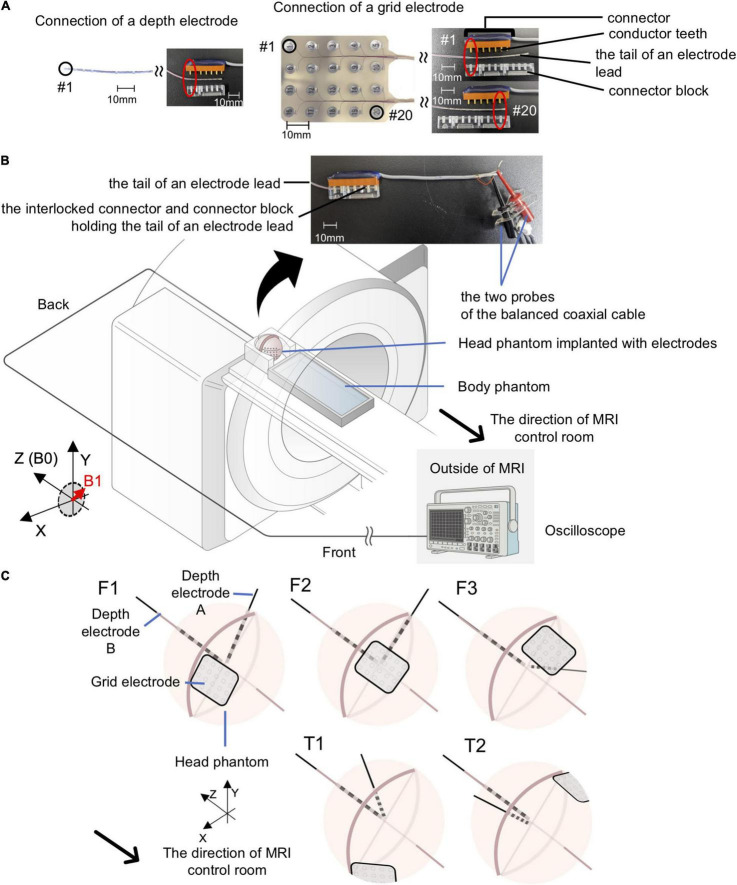
Experimental setup for gradient switching-induced voltage measurement. **(A)** Modified Ad-Tech connector cables and the electrodes. An Ad-Tech connector cable was cut at 10 cm away from the connector and 2 cm of the cable outer sheath was removed to expose the wires embedded within the cable. Each wire corresponds to one pair of conductor tooth on the connector; connection to an electrode contact can be established by connecting the probe of the balanced coaxial cable to one of these wires. The electrode was connected to the connector via a connector block; connection to any of the contacts on a grid electrode can be achieved by adjusting the relative position of the conductor tooth (on the connector) and the connector block. **(B)** Layout of the phantoms and all of the equipment during experiments for voltage measurement. Each electrode was connected via a modified Ad-Tech connecter cable (as in A) and a custom-made balanced coaxial cable to the oscilloscope placed outside the magnetic resonance imaging (MRI) in the control room. The electrodes were secured using memory foam cushions (not shown) in the head coil. The portion of electrode leads outside the head coil and the connector were placed at the center of the MRI bore as far away from the head coil as possible and firmly secured using sandbags (not shown). **(C)** Electrode configurations for voltage measurements. Upper panel – electrode configuration emulating frontal lobe implantation on the right (F1), center (F2), and left (F3). Lower panel – electrode configuration emulating temporal lobe implantation on the right (T1) and left (T2). See Table 2 for electrode contact pairs used for the measurement. Only the Unique medical electrodes are shown for illustrative purpose.

Voltages were measured using a balanced coaxial probe connected to a 350-MHz digital oscilloscope (MDO3034; Tektronix Inc., Beaverton, OR, United States) placed in the MRI control room. The balanced coaxial probe consisted of two 10-m long 50-ohm coaxial cables (RG-58/U) in which the shields were soldered together, and a 950-ohm resistor attached in series to each cable’s end (AKA 20:1 ‘low impedance’ probe) (Smith, 1993; Lemieux et al., 1997). The two probes of the balanced coaxial probe were connected to the icEEG electrodes via a connector block and a 10-cm connector cable (Tech-Attach Connection System; Ad-Tech Medical) modified for the voltage measurement to ensure electrical isolation between electrode tails and between contacts (Figures 3A,B).

### Movement Measurement Methodology

**FIGURE 4:**
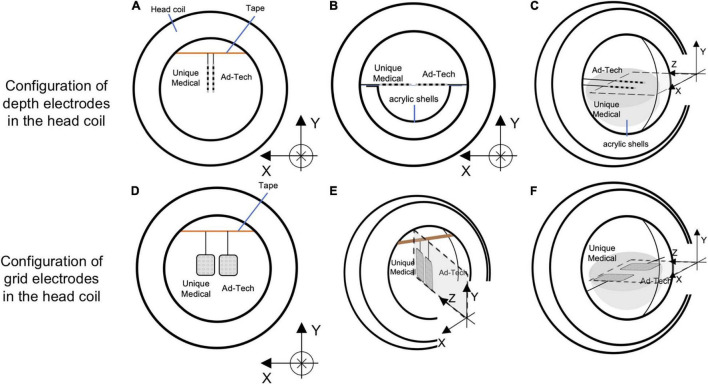
Configurations of the electrodes in the head coil during experiments for detecting gradient switching-induced electrode movement by visual assessment. Configurations of depth electrodes were shown in the upper panel and grid electrodes in the lower panel. Two depth electrodes (one Unique Medical and another Ad-Tech) were placed parallel to the *Y*-axis **(A)**, *X*-axis **(B)**, or *Z*-axis **(C)**. Two grid electrodes (one Unique Medical and another Ad-Tech) were placed in the XY-plane **(D)**, YZ-plane **(E)**, or ZX-plane **(F)**. In **(A,D,E)**, the electrodes were hanged down from a surgical tape (indicated as orange line) that was placed across the head coil parallel to the *X*-axis, with the end taped to the head coil. Each electrode was taped to the surgical tape at a point 7.5 cm from the tip. In **(B,C,F)**, the electrodes were placed on an isometric graph paper attached on the acrylic shell (that was used for preparing the head phantom). In all **(A–F)**, the electrodes were placed as close as possible to the center of the head coil (without touching each other), which was then placed at the isocenter of the magnetic resonance imaging scanner bore while running scans of EPI sequence.

**FIGURE 5:**
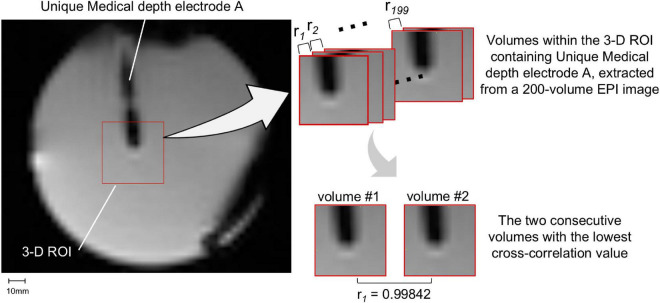
Movement measurement methodology – using image analysis. A three-dimensional (3D) region-of-interest (ROI) of 10 × 10 × 10 voxels containing either the tip of a depth electrode or contact #1 of a grid was extracted. Only Unique Medical depth electrode A and its 3D ROI are shown **(left panel)**. The cross-correlation value (*r*_*i*_) between two consecutive EPI volumes within the 3D ROI was calculated **(right upper panel)**: the lower the correlation value, the more dissimilar the pair of volumes were and thus the more likely the electrode was displaced. The pair of consecutive volumes with the lowest correlation coefficient were visually examined and any visible displacement of the electrode tip was physically measured. The two consecutive volumes within the 3D ROI containing Unique Medical depth electrode A with the lowest cross-correlation value was shown in the **right lower panel**.

We assessed the potential electrode movement induced by the magnetic field’s gradients switching during the scan using two different approaches: visual assessment and image analyses. For visual assessment, an echo-planar imaging (EPI) scan was acquired (see sequence parameters below) with the electrodes placed in the head coil without the gel head phantom because the gel was not completely transparent (Figure 4). For image analyses, we used one of the 200-volume EPI scan images acquired during the RF-induced heating experiments (experiment B as described in the section “*The Experiments: Electrode and phantom configurations”* below). We evaluated the displacement of the electrode tip between two consecutive EPI volumes using the following steps (Figure 5). (1) We identified the tip of each depth electrode and contact #1 of each grid electrode (see Figure 1B for the position). (2) For each electrode, we extracted a three-dimensional (3D) region of interest (ROI) of 10 × 10 × 10 voxels containing either the tip of a depth electrode or the contact #1 of a grid (voxel size of EPI = 3.7 × 3.7 × 3.7 mm). This resulted in five 3D ROIs, each containing one of the five electrodes implanted in the head phantom. (3) We calculated the cross-correlation between two consecutive EPI volumes within the 3D ROI to look for dissimilarity using an FSL tool (*fslcc*^1^). This was repeated for each 3D ROI extracted in step (2). Cross-correlation value was 1.00000 between two identical 3D ROI and the mean cross-correlation value among nine 3D ROIs extracted at random location from the EPI volume (resulted in nine dissimilar ROI-restricted EPI volumes) was 0.36396 (range 0.10098-0.84291). Thus, any cross-correlation value lower than 1.00000 indicates dissimilarity and possible electrode displacement. (4) For each 3D ROI, the pair of consecutive volumes with the lowest correlation value was considered the most dissimilar and identified as the pair with the greatest electrode displacement. We visually examined the tip of a depth electrode or contact #1 of a grid between the pair of consecutive volumes identified and physically measured the displacement if there was any.

### Scanning Sequences

All measurements were performed in a 3.0 T MRI scanner (GE 3 T MRI Signa Architect, No. EM0219; GE Medical Systems, Milwaukee, WI, United States) using the standard RF body transmit and head-receive coils, the latter with an opening at the back to allow the wires to pass straight to the back of the MRI bore. In RF-induced heating, switching gradient-induced voltage and electrode movement experiments described in the section “*The Experiments: Electrode and phantom configurations*” below, the parameters of the scanning sequences used were as follows: localizer (two-dimensional gradient-recalled steady state) acquired in sagittal, coronal, and axial separately {sagittal: echo time [TE] = 5.6 ms, repetition time [TR] = 20 ms, flip angle = 30°, field of view (FOV) = 28 cm, matrix = 256 × 256, five 5-mm-thick slices; coronal: TE = 7.6 ms, TR = 20 ms, flip angle = 30°, FOV = 28 cm, matrix = 256 × 256, five 5-mm-thick slices; axial: TE = 10 ms, TR = 20 ms, flip angle = 30°, FOV = 28 cm, matrix = 256 × 256, five 5-mm thick slices}; EPI (T2*-weighted gradient-recalled echo planar, TE = 22 ms, TR = 1900 ms, flip angle = 70°, FOV = 23 cm, matrix = 64 × 64, 33 3.7-mm-thick slices) acquired as a 200-volume image per scan (total scan time = 6 min 20 s) and scans were repeated if required; magnetization-prepared rapid gradient-echo (MP-RAGE) (3D T1-weighted inversion recovery, TE = 2.5 ms, TR = 2500 ms, flip angle = 9°, FOV = 25.6 cm, matrix = 256 × 256 × 166, 1-mm-thick slices); and fast spin echo (FSE) (two-dimensional T2-weighted spin echo, TE = 102 ms, TR = 4000 ms, flip angle = 180°, FOV = 22 cm, matrix = 512 × 256, 24 5-mm-thick slices).

### The Experiments: Electrode and Phantom Configurations

Since the mechanism underlying RF-induced heating and switching gradient-induced voltage differs (see below), the electrode and phantom were configured differently for temperature and voltage measurements to maximize the effects of each factor (worst-case scenarios). Table 2 summarizes the MRI sequences tested in each experiment below.

**TABLE 2:** MRI sequences tested in the radio frequency-induced heating, switching gradient-induced voltage and electrode movement experiments.

Experiments	Measurement #	MRI sequences tested
**Radio frequency-induced heating** • Only low B_1+_RMS or SAR sequences (localizer, EPI, MP-RAGE) were used when the EEG amplifiers were placed in the scanner. These are the sequences required to complete a typical icEEG-fMRI experiment at our center. • A high B_1+_RMS sequence or SAR (FSE) that causes substantial heating was used as a positive control, after the amplifiers were removed from the scanner. This sequence would not be used during a typical icEEG-fMRI experiment.		

Experiment A – shorter recording (separate scan of each MRI sequence)		
Configuration A1 – electrodes oriented perpendicular to B_0_	A1.1	EPI
	A1.2	MP-RAGE
	A1.3	FSE
Configuration A2 – electrodes oriented parallel to B_0_	A2.1	EPI
	A2.2	MP-RAGE
	A2.3	FSE
Experiment B – longer recording (continuous scan of all MRI sequences)	B	Localizer + 10 consecutive EPI scans + MP-RAGE

**Switching gradient-induced voltage** • Nerve stimulation is likely caused by induced currents and voltages resulted from a time-varying gradient magnetic field of less than 10 kHz.		
	See Table 4	EPI

**Electrode movement** • Rapidly switching gradient field may cause rapid movement of implants because implants are subjected to mechanical force when exposed to gradient field.		
	See Figure 4	EPI

*B_1+_RMS, root mean square value of B_1+_ averaged over a period of 10 s; EPI, echo-planar imaging; FSE, fast spin echo; icEEG-fMRI, simultaneous acquisition of icEEG and fMRI; MP-RAGE, magnetization-prepared rapid gradient-echo; MRI, magnetic resonance imaging; SAR, specific absorption ratio.*

#### Radio Frequency-Induced Heating

For temperature measurements, since the RF-induced heating may vary depending on the orientation of the electrodes relative to the scanner static magnetic field (B_0_) (Nordbeck et al., 2008; Winter et al., 2020), temperature change was first recorded during a scan of each MRI sequence to identify the worst case (experiment A, shorter recording), and then recorded during a scan using all MRI sequences over a total scan period of approximately 70 min (experiment B, longer recording), to mimic a typical icEEG-fMRI acquisition. In experiment A, measurements were performed using two configurations as follows: electrodes oriented perpendicularly to B_0_ (A1) and electrodes parallel to B_0_ (A2). Experiment B was performed using the configuration found in experiment A that resulted in most heating (which corresponded to A1, see Table 3) with MRI sequences in the following order: localizer, EPI (200-volume image × 10, total scan time approximately 63 min), and MP-RAGE (scan time ∼8 min). Experiment B was repeated three times for Unique Medical electrodes. Repeated measurement has been well-documented previously using Ad-Tech electrodes (Boucousis et al., 2012) and thus not repeated here.

**TABLE 3:** Results of Experiment A.

		Depth	Grid	Control
Measurement	B_1+_RMS	△T(°C)	△T(°C)	△T(°C)
**Configuration A1: Orthogonal**				
A1.1 (EPI)	0.77	0.2	0.1	0.1
A1.2 (MP-RAGE)	0.72	0.1	0.1	0.1
A1.3 (FSE)	2.57	1.9	0.7	0.2
**Configuration A2: Parallel**				
A2.1 (EPI)	0.77	0.1	0.1	0.1
A2.2 (MP-RAGE)	0.72	0.1	0.1	0.1
A2.3 (FSE)	2.57	0.6	0.4	0.1

*Temperature measurements for the Unique Medical electrodes.*

*EPI, echo-planar imaging; MP-RAGE, magnetization-prepared rapid gradient-echo; FSE, fast spin echo; B_1+_RMS, root mean square value of B_1+_ averaged over a period of 10 s; Depth, depth electrode; Grid, grid electrode; △ T, maximum change in temperature.*

#### Switching Gradient-Induced Voltage

For voltage measurements, since the gradient field amplitude varies as a function of location along the scanner’s long axis (Schaefer et al., 2000) and to maximize the effect (worst case scenarios), measurements were performed with electrodes configured in orientations mimicking either frontal lobe or temporal lobe implantations (see Table 4 and Figure 3C). For frontal lobe implantation configurations, electrodes were placed to mimic either implantation in the right (configuration F1), center (configuration F2), or left (configuration F3) aspects of the frontal lobe. Two temporal lobe implantation configurations were used: right (configuration T1) and left (configuration T2).

**TABLE 4:** Electrode configurations for voltage measurement.

Configuration	Location	Voltage measurement #	First contact (see Figure 1B for electrode type and contact numbering)	Location of the first contact in relation to the phantom	Second contact (see Figure 1B for electrode type and contact numbering)	Location of the first contact in relation to the phantom
**Frontal lobe**						
F1	Right	F1.1	Grid #1	Inner surface	Grid #20	Inner surface
		F1.2	Grid #1	Inner surface	Depth A #1	Near center
F2	Middle	F2.1	Grid #1	Inner surface	Grid #20	Inner surface
F3	Left	F3.1	Grid #1	Inner surface	Grid #20	Inner surface
		F3.2	Grid #1	Inner surface	Depth A#1	Near center
**Temporal lobe**						
T1	Right	T1.1	Grid #1	Inner surface	Grid #20	Inner surface
		T1.2	Grid #1	Inner surface	Depth A #1	Near center
		T1.3	Depth A #1	Near center	Depth B #1	Near center
T2	Left	T2.1	Grid #1	Inner surface	Grid #20	Inner surface
		T2.2	Grid #1	Inner surface	Depth A #1	Near center
		T2.3	Depth A #1	Near center	Depth B #1	Near center

*Grid, grid electrode; Depth, depth electrode; Depth A, the depth electrode that was implanted quasi-perpendicular to the grid electrode; Depth B, depth electrode that was implanted quasi-parallel to the grid (see Figures 1A,C).*

According to Maxwell’s equation, the larger the circuit within the electrodes and leads perpendicular to the magnetic field, the larger the induced voltage (Georgi et al., 2004). Based on this equation and previous studies (Carmichael et al., 2010; Boucousis et al., 2012), we measured the gradient-induced voltage between two most distant electrode contacts aiming at maximizing the loop area (representing the worst-case condition). The measurement was performed with either of the following electrode contact combinations: between contact #1 and #20 of a grid electrode, between contact #1 of a depth electrode and contact #1 of a grid electrode, or between contact #1 of two depth electrodes (the third combination was only available for depth electrodes from Unique Medical). Electrodes that were not used during each measurement were electrically shorted at the tails/cable terminations. The last four columns of Table 4 summarize the electrode contact combinations used in each configuration and the location of each electrode contact in relation to the phantom. The orientation of each electrode contact relative to the MRI bore axis is shown in Figure 3C.

#### Electrode Movement

For visual assessment of electrode movement, the electrodes were oriented either parallel to the *X-, Y*- or *Z*-axis of the MRI bore (Figure 4). For the orientation parallel to the *Y*-axis configuration, the electrodes were fixed at a point 75 mm away from the tip of a depth electrode or the distal edge of a grid electrode using a surgical tape; the tip or edge of the electrodes was hanging freely. An isometric graph paper was placed at the back to facilitate detection of any possible movement, without touching the electrodes. For the orientation parallel to the *X*- or *Z*-axis configuration, the electrodes were placed on a piece of isometric graph paper, held at the same point as above-mentioned. Movement was assessed under both direct visual observation and through video recording taken during the experiment.

## Results

### Temperature Measurements

**FIGURE 6:**
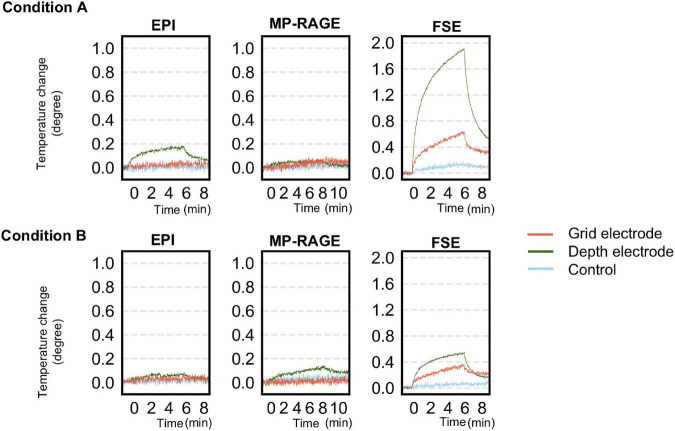
Temperature changes observed during a scan of each sequence for simultaneous acquisition of icEEG and fMRI (EPI, MP-RAGE), and a high-SAR sequence (FSE) as control in experiment A. The **upper panel** shows the changes at each electrode when oriented perpendicularly to B_0_ (configuration A1). The **lower panel** shows the changes at each electrode when oriented parallel to B_0_ (configuration A2). The temperature change was generally larger in configuration A1 except for MP-RAGE. SAR, specific absorption rate; EPI, echo planar imaging; FSE, fast spin echo; MP-RAGE, magnetization-prepared rapid gradient-echo.

**FIGURE 7:**
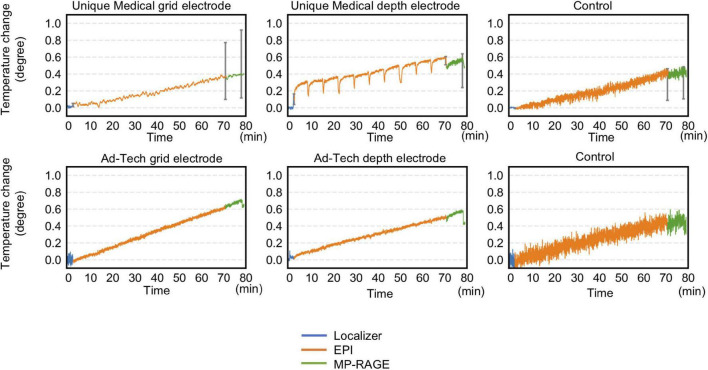
Temperature changes throughout the 70-min acquisition using all sequences of a typical simultaneous acquisition of icEEG and fMRI experiment. Radio frequency-induced heating on grid and depth electrodes placed orthogonal to the static magnetic field were shown. For Unique Medical electrodes, each trace represents the median temperature changes from three measurements. Error bars show the interquartile range of the measured temperature at the end of each sequence. The total temperate increase was within 1°C regardless of electrode type. Compared to Ad-Tech, Unique Medical electrodes showed a more abrupt temperature decrease between images.

Table 3 and Figure 6 summarize temperature changes for each sequence used in our icEEG-fMRI protocol, and a high specific absorption rate (SAR) sequence (FSE). For configuration A1, the observed maximum temperature increases were 0.2, 0.1, and 1.9°C during EPI, MP-RAGE, and FSE, respectively, for the depth electrode; and 0.1, 0.1, and 0.7°C during EPI, MP-RAGE, and FSE, respectively, for the grid electrode. For configuration A2, the observed maximum temperature increases were 0.1, 0.1, and 0.6°C during EPI, MP-RAGE, and FSE, respectively, for the depth electrode; and 0.1, 0.1, and 0.4°C during EPI, MP-RAGE, and FSE, respectively, for the grid electrode. Figure 7 shows the results of Experiment B. For Unique Medical electrodes, the observed total median temperature increases were 0.4 and 0.6°C for the grid and depth electrodes, respectively. For Ad-Tech electrodes, the observed total temperature increases were 0.7 and 0.6°C for grid and depth electrodes, respectively. The temperature increased monotonically with time at all electrodes throughout the course, although a more rapid increase on depth electrodes was observed upon the start of MP-RAGE.

### Voltage Measurements

Under frontal lobe implantation configurations, the greatest mean gradient-induced voltage was 43.5 (standard deviation, SD 0.5) mV for Unique Medical and 64.0 (SD 1.4) mV for Ad-Tech electrodes. Under temporal lobe implantation configurations, the greatest mean gradient-induced voltage was 79.3 (SD 1.6) mV for Unique Medical and 86.6 (SD 1.1) mV for Ad-Tech electrodes. A larger voltage was induced under the left temporal lobe implantation configurations. The results are shown in Table 5.

**TABLE 5:** Observed gradient-induced voltages.

Configuration	Measurement #	Peak voltage (mV)			
		
		Unique Medical		Ad-Tech	
			
		Mean	*SD*	Mean	*SD*
F1	F1.1	43.5	0.5	14.6	0.9
	F1.2	7.2	1.0	55.7	0.6
F2	F2.1	22.3	0.4	64.0	1.4
F3	F3.1	29.0	0.9	14.6	0.8
	F3.2	26.0	0.5	21.1	1.3
T1	T1.1	55.5	1.0	66.3	0.9
	T1.2	19.6	0.9	51.8	1.1
	T1.3	41.5	0.6	NA	
T2	T2.1	64.9	5.1	10.7	0.4
	T2.2	55.9	1.8	86.6	1.1
	T2.3	79.3	1.6	NA	

*Depth, depth electrode; Grid, grid electrode; NA, not applicable.*

*Depth A, the depth electrode that was implanted quasi-perpendicular to the grid electrode; Depth B, the depth electrode that was implanted quasi-parallel to the grid electrode (see Figures 1A,C).*

*Mean and standard deviation of 10 peak voltages were shown. Note that only one Ad-Tech depth electrode was implanted in the phantom, and thus the voltage measurement was not applicable for the combination of the two Ad-Tech depth electrodes. NA, not applicable; SD, standard deviation.*

### Movement Measurements

Under visual assessment, no movement was detected under all tested conditions. Using EPI image analyses, the lowest cross-correlation value of each 3D ROI containing a depth electrode tip or grid electrode contact #1 was shown in Table 6. Visual inspection revealed no displacement even between the volume pairs with the lowest cross-correlation value (see Figure 5 for an example). The variation in cross-correlation values were due to slight changes in global intensity between EPI volumes instead of a visible displacement.

**TABLE 6:** The lowest cross-correlation value between two consecutive EPI images acquired during a 200-volume EPI scan.

Electrode	The lowest cross-correlation value	The corresponding pair of EPI volumes (image #)
Unique Medical grid electrode contact #1	0.99996	1/2
Unique Medical depth electrode A tip	0.99912	1/2
Unique Medical depth electrode B tip	0.99842	1/2
Ad-Tech grid electrode contact #1	0.99994	1/2
Ad-Tech depth electrode A tip	0.99976	1/2

*Depth electrode A, the depth electrode that was implanted quasi-perpendicular to the grid electrode; depth electrode B, the depth electrode that was implanted quasi-parallel to the grid electrode (see Figures 1A,C). EPI, echo-planar imaging.*

## Discussion

This study addressed the safety of performing icEEG-fMRI at 3 T using depth and grid electrodes available in Japan, under conditions tested in this study. For all conditions tested, the gradient-induced voltages were within 100 mV and the maximum temperature increase was within 1°C, both fulfilling the criteria according to current safety standards (Georgi et al., 2004; IEC, 2015). A prolonged acquisition that lasted approximately 70 min under the worst-case scenario also did not result in a temperature increase exceeding 1°C in the vicinity of the electrodes. Despite the difference in the combination of electrodes and scanner used in our study, the results were comparable to studies reported on the feasibility of icEEG-fMRI. For example, Carmichael et al. (2010) and Boucousis et al. (2012) reported temperature changes within 1°C, induced voltages within 100 mV and no significant implant movements using Ad-Tech electrodes in a 1.5 T Siemens scanner and a 3 T GE scanner, respectively. The findings of our study may serve as a guidance for safety precaution to centers intending to perform MRI imaging for post-implantation localization of icEEG electrodes. MP-RAGE sequence is rather safe but not high SAR sequence such as FSE; however, a local safety protocol should be developed in each center because a slight difference in MRI scanner, coils, electrode and leads configuration may result in considerable differences in the safety profile of implants in an MRI (Carmichael et al., 2008).

Radio frequency-induced heating can result from implanted electrodes acting as a resonating linear antenna. The high electrical resistance of the tissue causes local resistive heating and increases temperature (Mattei et al., 2008). RF-induced heating can cause neuronal damage when prolonged increases of 5°C above body temperature occur (Dewhirst et al., 2003; Georgi et al., 2004; Boucousis et al., 2012). Current safety standards have further limited the acceptable heating of a device more conservatively to within 1°C (IEC, 2015). In our experiments, the temperature increase during the prolonged 70-min acquisition was well below this limit. Our observations provide evidence that the risk of excessive heating is manageable in the specific circumstances tested; namely a 3 T GE MRI scanner with body RF coil, grid, and depth electrodes configured to mimic implantation in the frontal and temporal lobes. We found that RF-induced heating was more prominent with the electrodes placed orthogonal to the static magnetic field. Previous studies have shown that the closer the distance between an electrode and the RF transmitter coil, the higher the temperature of the induced heating (Mattei et al., 2008; Bhusal et al., 2018). For this reason, the temperature increased more when an electrode was placed orthogonal, in which the electrode has been inserted from the side and thus closer to the transmitter coil. These observations along with ours are in line with the observations of some early case reports on the adverse effects potentially resulting from RF-heating in the MRI. These include a transient dystonic and ballistic movements following a head MRI (Spiegel et al., 2003) and a peri-electrode hemorrhage following a lumbar spine MRI (Henderson et al., 2005) both performed at 1.0 T on patients with Parkinson’s disease that was implanted with bilateral DBS. The difference in severity of the adverse effects in these reports suggested the potential impact of electrodes’ orientation, length, and configuration on RF-induced heating. Indeed, a study on a cardiac pacemaker implant showed the impact of lead pathway and device position on RF-heating during MRI (Nordbeck et al., 2009).

Gradient switching during EPI causes polarity of the magnetic field to change rapidly and results in an induced current. The induced current is dependent on the frequency and the cross-sectional area of the electrode contacts. In general, stimuli above 10 kHz such as those generated by RF pulse do not evoke action potentials in neuronal cells (Patrick Reilly, 2016; Ziegelberger et al., 2020). Therefore, only the effect of gradient switching below 10 kHz are considered in terms of induced voltage that could result in inadvertent neuronal stimulation or brain tissue damage, which has been suggested to occur at voltage exceeding 100 mV (Georgi et al., 2004). In this study, neither implantation mimicking frontal nor temporal lobe exceeded this limit, thus confirming the safety of icEEG-fMRI using these implantation schemes, which predominate at our center.

Implants such as intracranial electrodes can be subjected to mechanical force when exposed to magnetic gradient, depending on their orientation. The resultant force may lead to movement of implants against the surrounding tissue (Erhardt et al., 2018). Our study showed that gradient switching during the EPI sequence did not cause any visible movement by direct visual inspection and analysis of the EPI images. We did not perform a formal measurement of static magnetic field-induced forces according to the American Society for Testing of Materials because these were previously reported to be insignificant for non-ferromagnetic platinum-iridium electrode contacts (Carmichael et al., 2010; Boucousis et al., 2012).

This study showed that the total temperature increase during a 70-min acquisition that included all MRI sequences used in a typical icEEG-fMRI experiment at our center was below the established safety limit of 1°C. Nevertheless, as the increase was summative following each scan, a sufficient interval should be placed in between scans if more scans are needed. We did not evaluate the difference in electrode heating between the left and right side of the scanner because our MRI scanner uses multiple RF transmissions in parallel (multidrive RF transmission technology) that minimizes the RF non-uniformity especially around the isocenter, where the electrodes were placed. Therefore, our findings should not be extrapolated to MRI scanners without this technology. The minimal increase in temperature recorded in this study may well be attributed to the configuration of the connecting cables placed along the central axis of the coil because the closer the cable is placed to the transmitter coil, the more heat is generated (Carmichael et al., 2010; Bhusal et al., 2018). Although we did not evaluate the effect of cable length in this study, it should also be carefully considered and optimized to the strength of the magnetic field; the temperature increase is greatest when the cable length is one fourth or half the RF wavelength (Yeung et al., 2007). Although we expect the effect of brain perfusion to mitigate RF-induced heating in living human subjects and therefore our heating measurements can be taken to reflect a worst-case scenario in this specific sense, the utmost care and attention to detail is recommended when considering performing icEEG-fMRI in patients; specifically, a local safety assessment and experiments such as those presented here, are recommended as a minimum. Our findings are not generalizable to other MRI scanners, RF-transmit coils, or electrodes from other manufacturers (Carmichael et al., 2008).

## Data Availability Statement

The original contributions presented in this study are included in the article, further inquiries can be directed to the corresponding author.

## Author Contributions

YF and HMK were responsible for the conception and design of the study under the advice of LL. YF, HMK, MH, MK, YK, and HT were responsible for data acquisition. YF and HMK were responsible for data analyses, drafting a significant portion of the manuscript and figures. YF conducted the statistical analyses under the supervision of HMK. All authors were responsible for interpreting the findings, contributed toward subsequent revisions, and approved the submitted manuscript.

## Conflict of Interest

The authors declare that the research was conducted in the absence of any commercial or financial relationships that could be construed as a potential conflict of interest.

## Publisher’s Note

All claims expressed in this article are solely those of the authors and do not necessarily represent those of their affiliated organizations, or those of the publisher, the editors and the reviewers. Any product that may be evaluated in this article, or claim that may be made by its manufacturer, is not guaranteed or endorsed by the publisher.

## Footnotes

1
https://fsl.fmrib.ox.ac.uk/
